# Comprehensive analysis of somatic mutator-derived and immune infiltrates related lncRNA signatures of genome instability reveals potential prognostic biomarkers involved in non-small cell lung cancer

**DOI:** 10.3389/fgene.2022.982030

**Published:** 2022-09-26

**Authors:** Cai-Zhi Yang, Ting Yang, Xue-Ting Liu, Can-Feng He, Wei Guo, Shan Liu, Xiao-Hui Yao, Xi Xiao, Wei-Ran Zeng, Li-Zhu Lin, Zhong-Yu Huang

**Affiliations:** ^1^ The First School of Medicine, Guangzhou University of Chinese Medicine, Guangzhou, China; ^2^ Department of Oncology, The First Affiliated Hospital of Guangzhou University of Chinese Medicine, Guangzhou, China; ^3^ Guangzhou University of Chinese Medicine, Guangzhou, China; ^4^ Oncology Department, The First Affiliated Hospital of Guangzhou Medical University, Guangzhou, China; ^5^ Guangzhou First People’s Hospital School of Medicine, South China University of Technology, Guangzhou, China; ^6^ Formula-Pattern Research Center, School of Traditional Chinese Medicine, Jinan University, Guangzhou, China

**Keywords:** non-small cell lung cancer, long non-coding RNA, genomic instability, immune infiltration, prognosis

## Abstract

**Background:** The function and features of long non-coding RNAs (lncRNAs) are already attracting attention and extensive research on their role as biomarkers of prediction in lung cancer. However, the signatures that are both related to genomic instability (GI) and tumor immune microenvironment (TIME) have not yet been fully explored in previous studies of non-small cell lung cancer (NSCLC).

**Method:** The clinical characteristics, RNA expression profiles, and somatic mutation information of patients in this study came from The Cancer Genome Atlas (TCGA) database. Cox proportional hazards regression analysis was performed to construct genomic instability-related lncRNA signature (GIrLncSig). Gene Ontology (GO) and Kyoto Encyclopedia of Genes and Genomes (KEGG) enrichment analyses were performed to predict the potential functions of lncRNAs. CIBERSORT was used to calculate the proportion of immune cells in NSCLC.

**Result:** Eleven genomic instability-related lncRNAs in NSCLC were identified, then we established a prognostic model with the GIrLncSig ground on the 11 lncRNAs. Through the computed GIrLncSig risk score, patients were divided into high-risk and low-risk groups. By plotting ROC curves, we found that patients in the low-risk group in the test set and TCGA set had longer overall survival than those in the high-risk group, thus validating the survival predictive power of GIrLncSig. By stratified analysis, there was still a significant difference in overall survival between high and low risk groups of patients after adjusting for other clinical characteristics, suggesting the prognostic significance of GIrLncSig is independent. In addition, combining GIrLncSig with TP53 could better predict clinical outcomes. Besides, the immune microenvironment differed significantly between the high-risk and the low-risk groups, patients with low risk scores tend to have upregulation of immune checkpoints and chemokines. Finally, we found that high-risk scores were associated with increased sensitivity to chemotherapy.

**Conclusion:** we provided a new perspective on lncRNAs related to GI and TIME and revealed the worth of them in immune infiltration and immunotherapeutic response. Besides, we found that the expression of AC027288.1 is associated with PD-1 expression, which may be a potential prognostic marker in immune checkpoint inhibitor response to improve the prediction of clinical survival in NSCLC patients.

## Introduction

As reported by the Global Cancer Statistics 2020, lung cancer (LC) has maintained a high incidence and mortality rate worldwide over the past decade (Sung et al., 2021). Among LC, non-small cell lung cancer (NSCLC) has the highest incidence rate of nearly 85%, and occurs particularly in non-smoking people, women and Asians. Rapid development and widespread availability of targeted therapy and immunotherapy in the field of NSCLC have improved the prognosis and prolonged life of patients with advanced diseases (Alexander et al., 2020). For this reason, Programmed death 1 (PD-1) or programmed death-ligand 1 (PD-L1) immune checkpoint inhibitors (ICIs) were recommended as the standard first-line therapy for most advanced NSCLC patients based on data from clinical studies (Borghaei et al., 2015; Antonia et al., 2017; Rittmeyer et al., 2017; Gandhi et al., 2018). Objective response rates to ICIs in patients with NSCLC range from 17% to 21%, and major responses were durable (Topalian et al., 2012; Garon et al., 2014). However, understanding the key molecules for the efficacy of PD-(L) 1 inhibitors is one of the most important challenges. In addition, an objective response rate of 29% was observed in the group having a higher tumor mutational burden (TMB) in advanced solid tumor patients when receiving the PD-1 blockade pembrolizumab as monotherapy (Marabelle et al., 2020). Nevertheless, some non-responders to ICIs have also been reported in these patients, and during ICI-based immunotherapies potential adverse events as well as increased costs were inevitable. This emphasizes the great need for innovative biomarkers to explore the effectiveness of immunotherapy.

The hallmarks of cancer comprise ten biological capabilities, including maintaining proliferation signals, inducing angiogenesis, activating invasion and metastasis, promoting tumor-inflammation, achieving replicative immortality, and evading immune destruction, among others. Notably, the basis of them is GI (Hanahan and Weinberg, 2011). A large-scale prospective clinical observational study showed that GI leads to mutations, somatic copy number alterations, and epigenomic alterations that generate phenotypic variation and intratumoral heterogeneity (Bailey et al., 2021). GI is an important driver of cancer evolution as well as a predictor of poor prognosis in NSCLC (Jamal-Hanjani et al., 2017). Establishing the relationship between GI, degree of intratumor heterogeneity, and clinical outcome could improve prognostic prediction including treatment response, decipher antitumor response and drug resistance, and guide future patient stratification and combination therapy (Bailey et al., 2021). Although molecular driver events such as EGFR, TP53, KRAS, and BRCA1 mutations, as well as GI, are already reported to be associated with survival and drug resistance (Feng et al., 2015), clear perception of their relationship with the clinical outcomes remains to be an unmet need in NSCLC. GI and increased somatic TMB have been reported to be correlated with alterations in DNA damage response and repair (DDR) genes, possibly due to enhancement of immunogenicity of DDR genes by increasing tumor-specific neoantigen load (Rizvi et al., 2015; Mouw et al., 2017; Chae et al. 2019b). It has been suggested in the literature that alterations in DDR gene may not only boost tumor immune recognition also tumor targeting through the way of neoantigen-independent, for example, promoting innate immune *via* the mediation of stimulator of interferon genes pathway (Ablasser et al., 2013; Barber, 2015; Parkes et al., 2017). Furthermore, cumulative GI can generate tumor neoantigens which might trigger immune infiltrating cells and result in spontaneous anti-tumor immune effect (Desrichard et al., 2016).

Tumor immune microenvironment (TIME) consists mainly of myeloid cells, lymphocytes and some other innate immune cells. The study for comprehensive genomic and immunological characterization of Chinese NSCLC patients demonstrated that tumor-infiltrating lymphocytes (TILs) were obvious in NSCLC and played important roles in suppression and promotion of tumor development (Zheng et al., 2017; Zhang et al., 2019). Immune infiltrates, as the major constituent in the tumor microenvironment (TME), has been shown to affect tumor development and immunotherapeutic responses (Zhang and Zhang, 2020). A meta-analysis of 29 trials including 86 ,000 patients indicated that a high level of CD8^+^ cells infiltration was linked to better outcomes among LC patients (Geng et al., 2015). There is a certain link between GI and TIME in NSCLC, which may contribute to predict the prognosis and need more acquaintance.

Long non-coding RNAs (lncRNAs), a set of non-coding RNAs consisting of >200 nucleotides, is the modulator of GI from chromosomes to DNA bases in tumor development by affecting aneuploidy formation and telomere length, regulating chromatin loop structures and DNA damage and repair (Guo et al., 2021). LncRNA NORAD preserves genomic stability by segregating PUMILIO proteins and regulates both ploidy and chromosomal stability directly (Lee et al., 2016; Munschauer et al., 2018). The role of lncRNAs in different tumor has also received in-depth investigations. Some lncRNAs have been found to involve in promoting and inhibiting the LC cell growth (Zhang et al., 2016; Jiang et al., 2021b), for example, LNC CRYBG3 interacting with Bub3 resulted in aberrant mitosis which led to aneuploidy and then promoted the development of NSCLC (Guo et al., 2021b). For another, accumulating evidence suggests that lncRNAs exert important effects in several stages of tumor immunity from antigen release to killing cancer cells. (Yu et al., 2018). And multicellular functions of lncRNAs are very pervasive in the TIME *via* cell–cell interactions (Park et al., 2022). For illustration, NEAT1 and LUCAT1, which are implicated in poor prognosis in NSCLC, perform functional actions in diverse types of immune cells (Sun et al., 2016; Agarwal et al., 2020; Xing et al., 2021). The involvement of lncRNAs in the occurrence and development of LC have attracted extensive attention. More and more studies have explored lncRNA signatures associated with GI in lung cancer, suggesting that GI-associated lncRNAs might be molecular biomarkers of prognosis. However, the biological signatures that are both related to GI and TIME in NSCLC patients have rarely been reported in these studies.

In this study, we identify the somatic mutator-derived and immune infiltrates related lncRNA signatures of GI, exploring the association between lncRNA signatures, GI and TIME, with the purpose of predicting the clinical outcomes and evaluating the treatment in NSCLC patients more effectively.

## Materials and methods

### Data collection

We downloaded clinical characteristics, RNA expression profiles and somatic mutation of patients with lung adenocarcinoma (LUAD) and lung squamous carcinoma (LUSC) from The Cancer Genome Atlas (TCGA) database (https://portal.gdc.cancer.gov/). Transcriptome data were distinguished based on mRNA and lncRNA profiles. We downloaded 999 samples in total. In addition, we also collected somatic mutation profiles of 1,053 patients and clinical characteristics of 990 patients from TCGA database for further independent validation analysis. According to the sample names, we matched data from these three components and removed patients with no crucial clinical factors or with a survival time of less than 1 month. As a result, further analysis was conducted on 418 samples. Finally, a total of 950 samples with complete clinical features, RNA expression profiles and somatic mutation information were retained for our analysis. Using annotations from GENCODE (http://www.gencodegenes.org), lncRNA and mRNA transcript profiles were generated. Since all data were obtained from the TCGA database and are open to the public, there was no need to publish an informed consent form.

### Identification of genomic instability-associated long non-coding RNAs

To identify genomic instability-associated lncRNAs, we arranged patients in order of most to least somatic mutations. Patients in the top 25% were referred to as the genomic instability (GU) group (*n* = 253) and the last 25% as the genomic stability (GS) group (*n* = 231). We used significance analysis of limma R package (3.52.2) to compare the expression of lncRNAs between the two groups, differentially expressed lncRNAs (fold change greater than 1.5 or less than -1.5, adjusted *p* < 0.05) were defined as genomic instability-associated lncRNAs. According to the expression levels of the GI-related lncRNAs, patients were classified into two clusters: the genomically stable-like (GS-like) cluster and the genomically unstable-like (GU-like) cluster by the hclust function in R software (4.2.0). We made comparisons of the somatic mutation counts and the expression of cancer biomarkers in both clusters by the Mann-Whitney U test. Statistical criteria were considered as *p* value < 0.05.

### Functional enrichment analysis

To predict the potential functions of GI-related lncRNAs, we linked the mRNA and lncRNAs by calculating the Pearson correlation coefficient using “limma” package (3.52.2) of R software. The top 10 mRNAs most correlated with each GI-related lncRNA were selected as target genes to construct a lncRNA-mRNA co-expression network. Using the “clusterProfiler” package (4.4.4) in R/Bioconductor, we performed Gene Ontology (GO) and Kyoto Encyclopedia of Genes and Genomes (KEGG) enrichment analyses on mRNAs. Statistical criteria were considered as adjusted *p* value < 0.05.

### Establishment of genomic instability- related long non-coding RNAs signature

After combining the RNA expression data of GI-related lncRNAs with survival time, all patients were assigned to train set and test set randomly, and compared the clinical features of the two sets. Cox proportional hazards regression analysis performed by the “survival” package (3.4–0) was used to analyze the relevance between GI-related lncRNAs expression and overall survival. According to the prognosis-related lncRNAs found in the train set, genomic instability-related lncRNA signature (GIrLncSig)was identified as prognostic model. The formula for calculating the GIrLncSig risk score was as follows:

$$GIrLncSig\;risk\;score=\overset{n}{\underset{i=1}{\sum }}Coefficient\;{\left(lncRNA\right)}_{i}\times \exp{\left(lncRNA\right)}_{i}$$

Where “Coefficient (lncRNA)_i_,” 1br of interferon genes pathway. “exp (lncRNA)_i_,” and “n” represent the estimated multivariable Cox regression coefficient, expression level, and the number of prognostic lncRNAs, respectively. According to this formula, the risk scores of all samples in the train, test, and the TCGA sets were hence calculated. The median score of the samples in the train set was used as a risk cutoff to classify all patients into the high-risk group with high GIrLncSig or low-risk group with low GIrLncSig.

### Correlation analysis of genomic instability-related long non-coding RNA signature with tumor immune infiltration

Patients with scores above the median value of the risk score in the train set were assigned to high-risk groups and vice versa to low-risk groups. Next, the level of 22 immune cell infiltrates in NSCLC cancer samples was quantified using the “CIBERSORT” software package based on RNA profiles with a cut-off *p* value <0.05. Further analyses explored the relationships between prognostic risk, somatic mutation and immune cell infiltration level.

### Performance validation of the long non-coding RNA signature

As a prognostic model, genomic instability-associated lncRNA signature was analyzed and validated for performance by a range of methods. First, Kaplan-Meier curves and log-rank test were used to evaluate overall survival (OS). Statistical criteria were considered as *p* value < 0.05. We further validated the applicability of the model by stratifying TCGA patients. The accuracy of the prognostic model was examined based on the area under the curve (AUC) using time-dependent receiver operating characteristic (ROC) curve analysis. With the GSE135222 dataset from the GEO database, external validation was also conducted to explore whether the lncRNAs in GIrLncSig could be applied in another independent dataset for OS prediction and response prediction to PD-1/PD-L1 inhibitors. Comparing our signature performance with other published signatures is done using ROC curves.

### Estimating the sensitivity of chemotherapy and molecular targeted drugs

The IC50 values and sensitivity of chemotherapy and molecular targeted therapy were compared between the high-risk group and the low-risk group through the “pRRophetic” package in R.

### Statistical analysis

We compared changes in categorical and quantitative data between groups using the Mann-Whitney U test. It is statistically significant when the two-tailed *p* < 0.05. R (4.2.0) executed all the statistical analysis.

## Result

### Landscape of somatic mutation in non-small cell lung cancer

**FIGURE 1:**
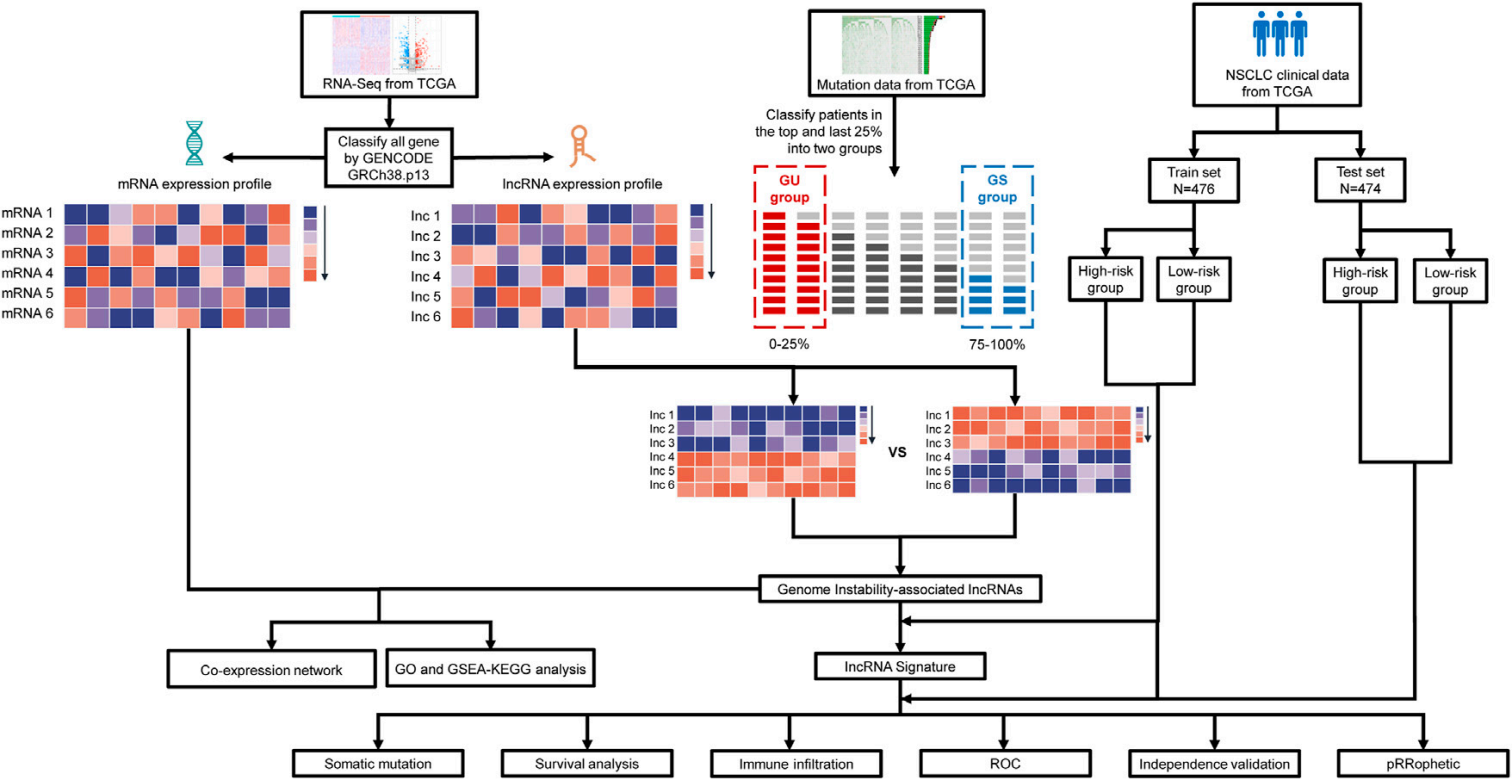
Diagram of the study’s flow. The TCGA database contains information on a large number of cancer patients, from which we obtain clinical characteristics, RNA expression profiles and somatic mutation information of NSCLC patients After identifying lncRNAs differentially expressed between patients in two groups with the top 25% and last 25% mutation counting frequencies, genomic instability-associated lncRNAs were obtained. Based on genomic instability-associated lncRNAs, we constructed an mRNA-lncRNA co-expression network and performed GO and KEGG functional enrichment analysis on the mRNAs in the network. Then genomic instability-related lncRNA signature (GIrLncSig) was established ground on relevant lncRNAs. Patients of train and test set were divided into two risk groups *via* the computed risk score. Relationship of risk score, immune infiltration and prediction of drug resistance were analyzed. Verification of the efficiency of the prognostic prediction was completed by survival analysis, ROC curves and independent validation. GU, genomically unstable; GS, genomically stable; TCGA, The Cancer Genome Atlas database; GO, Gene Ontology; GSEA, Gene Set Enrichment Analysis; KEGG, Kyoto Encyclopedia of Genes and Genomes; ROC, receiver operating characteristic.

**FIGURE 2:**
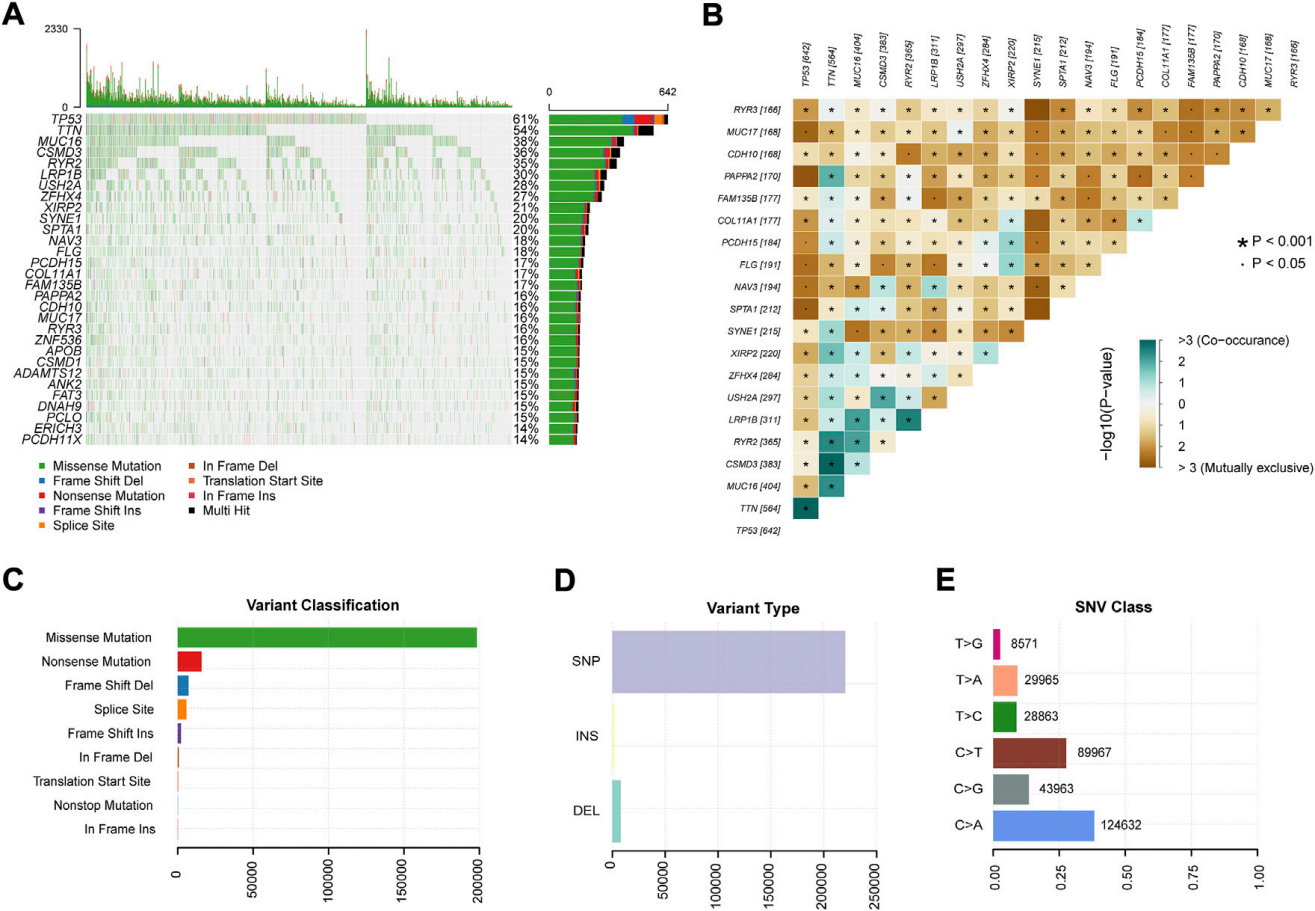
Summary of mutation profiles in NSCLC. **(A)** Waterfall plots showed the mutations for each gene in each sample. **(B)** Co-occurrence and mutual exclusion of genes with genomic alterations in NSCLC. **(C)** Missense mutations accounted for the largest proportion of the nine variant mutation forms in the sample. **(D)** SNPs were the most frequent of the three variant types in all samples. **(E)** C > A transition was the most frequent of the six subclasses of SNPs. SNP: single nucleotide polymorphism.

Figure 1 depicts the study design. We obtained genome-wide mutation files of 999 NSCLC patients from TCGA database. Somatic mutations existed in 977 (97.80%) NSCLC samples. Figure 2 summarized the somatic mutation information of all samples. Waterfall plot (Figure 2A) showed that TP53 mutated more frequently than any other genes accounting for 61%, followed by TTN (54%), MUC16 (38%), CSMD3 (36%), RYR2 (35%), LRP1B (30%), USH2A (28%), ZFHX4 (27%), XIRP2 (21%), and SYNE1 (20%). In Figure 2B, co-occurrence or mutually-exclusive expression of top 20 mutated genes was visualized. TTN was significantly concurrent with alterations in TP53 and CSMD3. As for gene variant classification, missense mutation was the most frequent alterations in 9 type variants (Figure 2C). Single nucleotide polymorphism (SNP) was the more frequent variant type than insertion or deletion (Figure 2D) and C>A alterations accounted mostly than other types of SNP (Figure 2E).

### Identification of long non-coding RNAs associated with genomic instability in non-small cell lung cancer

**FIGURE 3:**
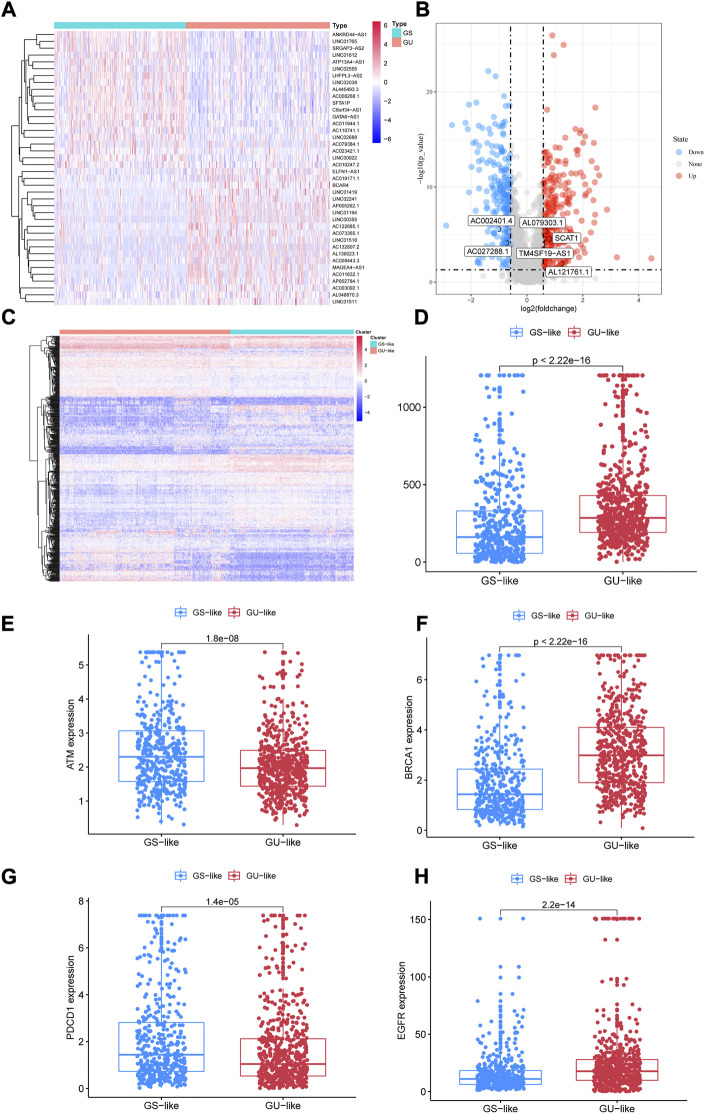
Identification of lncRNAs associated with genomic instability in NSCLC. **(A)** Differential AS events between GUL and GSL groups. **(B)** Volcano plot of 496 lncRNAs. **(C)** Heatmap of unsupervised hierarchical clustering analyses of 997 NSCLC patients. Red dots represent GUL clusters, blue dots represent GSL clusters. **(D)** The frequency of somatic cumulative mutations in GUL and GSL group. **(E–H)** ATM, BRCA1, PDCD1, and EGFR expression between the GSL group and GUL group. AS, alternative splicing; GUL, genomically unstable-like; GSL, genomically stable-like; GIrLncRNAs, genome instability-related lncRNAs.

After counting and ranking the somatic mutations in NSCLC samples, we defined the 25% of samples with the most somatic mutations (*n* = 253) as the genomic instability (GU) group and the 25% with the least (*n* = 231) as the genomic stability (GS) group. Using the limma R package, totally 496 lncRNAs with differential expression were selected as genomic instability-related lncRNAs (*p* < 0.05, Figures 3A,B). Among them, in GU group, the expression of 273 lncRNAs were upregulated, while the number of downregulated lncRNAs was 223 (Supplementary Table S1). Through unsupervised hierarchical clustering analysis, all NSCLC patients were clustered into the GS-like group and the GU-like group (Figure 3C). The frequency of somatic cumulative mutations in the GU-like group was evidently greater than the GS-like group (*p* < 0.001, Figure 3D). After that, we compared the expression of cancer biomarkers ATM, BRCA1, PDCD1, and EGFR gene in the two groups. In Figures 3E−H, the expressions of ATM and PDCD1 in the GU-like group were observably lower (*p* < 0.001), the expression was higher for BRCA1 and EGFR (*p* < 0.001). To sum up, the 496 lncRNAs could be recognized as candidate GU-lncRNAs.

### Construction of long non-coding RNA-mRNA Co-expression network and functional enrichment analysis

**FIGURE 4:**
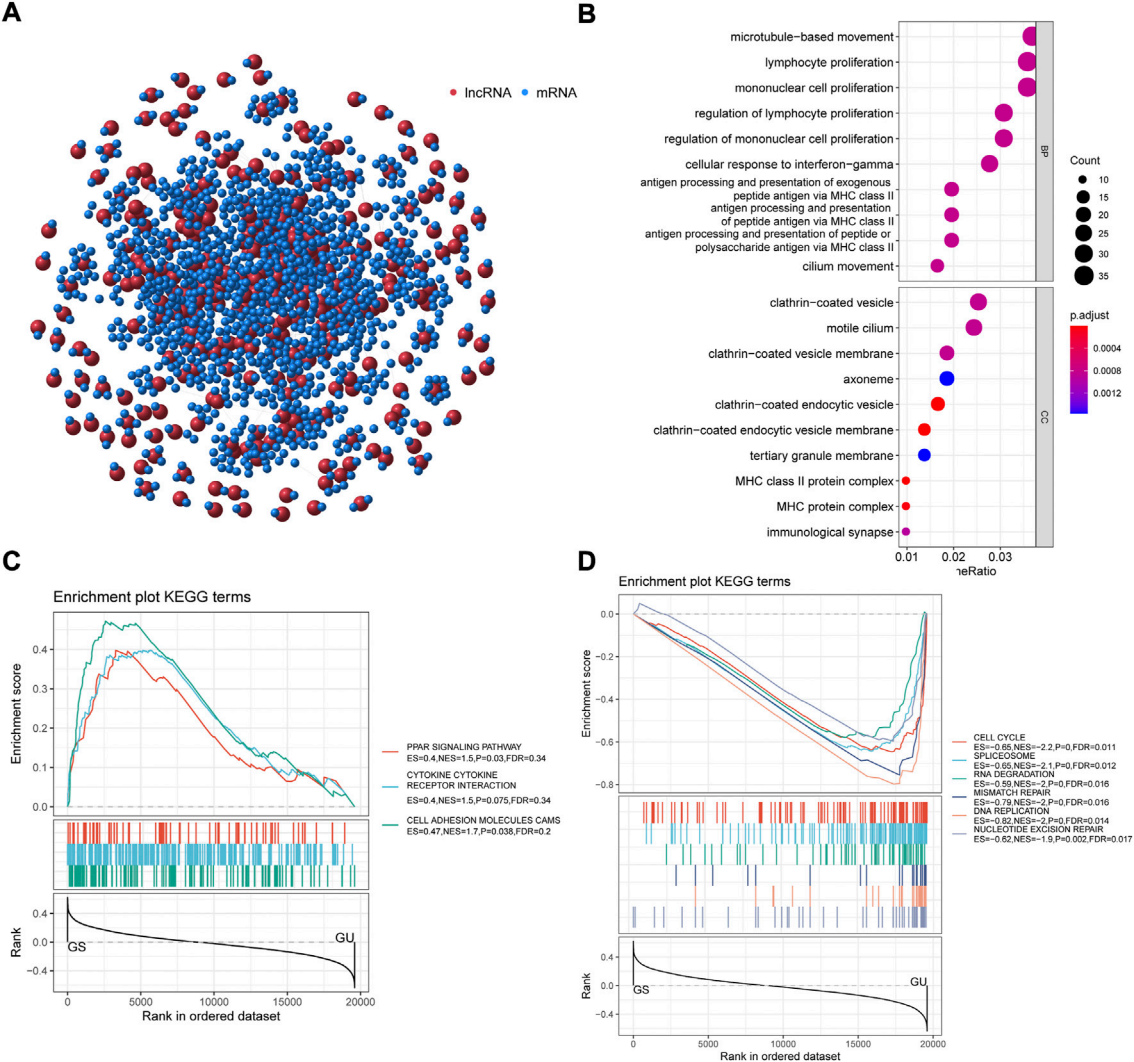
. lncRNA-mRNA co-expression network and functional enrichment analysis. **(A)** Co-expression network of mRNAs and lncRNAs, with red dots representing lncRNAs and blue dots representing mRNAs. **(B)** Bubble plot of GO analysis of differential AS events. **(C–D)** KEGG analysis plot of lncRNA-correlated PCGs. GO, gene ontology; KEGG, Kyoto Encyclopedia of Genes and Genomes; PCGs, protein-coding genes.

The top 10 protein-coding genes (PCGs) most associated with individual lncRNAs were identified as lncRNA-correlated PCGs by Pearson correlation coefficients. We constructed an lncRNA-mRNA co-expression network by linking PCG and lncRNA associated with lncRNA (Figure 4A). GO analysis revealed that correlated mRNAs are mainly enriched in the process of immune response, including lymphocyte proliferation, regulation of mononuclear cell proliferation, and cellular response to interferon gamma (Figure 4B). The result of KEGG-GSEA indicated that the PCGs of GS-like group were enriched in pathways associated with tumor microenvironment including PPAR signaling pathway, cytokine-cytokine receptor interaction, and cell adhesion molecules (CAMs), meanwhile PCGs of GU-like group were enriched in pathways associated with genomic instability consisting of cell cycle, homologous recombination, nucleotide excision repair and mismatch repair (Figures 4C,D). According to the GO and KEGG analysis results altered expression of lncRNA-mRNA co-expression network may affect the immune system leading to genomic instability in NSCLC.

### Exploration of 11-long non-coding RNA-based prognostic model related to genome instability

For the reason of investigating the potential prognostic significance of candidate GU-lncRNAs, 950 NSCLC patients screened by the TCGA database were randomized into the train set (*N* = 476) and test set (*N* = 474). Table 1 shows the clinical information of 950 patients in the TCGA database. The age at diagnosis was 90 years at the maximum and 33 years at the minimum, with a median age of 67 years. There were approximately 758 (79.79%) stage I-II patients and 192 (20.21%) stage III-IV patients. TP53 gene mutation occurred in 642 (67.58%) NSCLC patients. The baseline clinical data that includes age, gender, TNM stage, and TP53 mutation status of two NSCLC patients sets were comparable (*p* > 0.05, Table 1).

**TABLE 1:** Clinical information for three NSCLC patients sets in this study.

Covariates	Type	TCGA set	Training set	Testing set	*P*
		(*n* = 950)	(*n* = 476)	(*n* = 474)	
Age	≤65	414 (43.58%)	209 (43.91%)	205 (43.25%)	0.8892
	>65	536 (56.42%)	267 (56.09%)	269 (56.75%)	
Gender	FEMALE	378 (39.79%)	187 (39.29%)	191 (40.3%)	0.8013
	MALE	572 (60.21%)	289 (60.71%)	283 (59.7%)	
Stage	Stage I-II	758 (79.79%)	371 (77.94%)	387 (81.65%)	0.18
	Stage III-IV	192 (20.21%)	105 (22.06%)	87 (18.35%)	
T	T1-2	799 (84.11%)	408 (85.71%)	391 (82.49%)	0.2039
	T3-4	151 (15.89%)	68 (14.29%)	83 (17.51%)	
M	M0	918 (96.63%)	457 (96.01%)	461 (97.26%)	0.3751
	M1	32 (3.37%)	19 (3.99%)	13 (2.74%)	
N	N0	627 (66%)	309 (64.92%)	318 (67.09%)	0.5233
	N1-3	323 (34%)	167 (35.08%)	156 (32.91%)	
TP53 mutation status	With	642 (67.58%)	336 (70.59%)	306 (64.56%)	0.1573
	Without	308 (32.42%)	140 (29.41%)	168 (35.44%)	

After univariate Cox proportional hazard regression analysis, 36 lncRNAs from 496 candidate GU-lncRNAs were screened as prognostic-related lncRNAs which showed the greatest correlation with overall survival in NSCLC patients (*p* < 0.05, Supplementary Figure S1). Multivariate Cox proportional hazard regression analyses identified 11 of 36 candidate lncRNAs (SCAT1, AC002401.4, AL079303.1, AL121761.1, TM4SF19-AS1, AC027288.1, AC019117.3, AC079949.2, AC026369.3, AL355472.2, and MMP2-AS1) as independent prognostic lncRNAs (Table 2). Then a prognostic model of lncRNA signatures associated with genomic instability (GIrLncSig) was established from the coefficient of 11 genomic instability-related lncRNAs in multivariate Cox analysis and their expression level with the following calculation formula:

$$\begin{array}{ll} GIrLncSig\;risk\;score\\ & =\left(0.1743\times SCAT1\;expression\;level\right)\\ & +\left(0.0744\times AC002401.4\;expression\;level\right)\\ & +\left(-0.3116\times AL079303.1\;expression\;level\right)\\ & +\left(-0.0916\times AL121761.1\;expression\;level\right)\\ & +\left(0.1696\times TM4SF19-AS1\;expression\;level\right)\\ & +\left(-0.1710\times AC027288.1\;expression\;level\right)\\ & +\left(-0.0533\times AC019117.3\;expression\;level\right)\\ & +\left(0.0983\times AC079949.2\;expression\;level\right)\\ & +\left(-0.1876\times AC026369.3\;expression\;level\right)\\ & +\left(0.1164\times AL355472.2\;expression\;level\right)\\ & +\left(-0.1812\times MMP2-AS1\;expression\;level\right). \end{array}$$

**TABLE 2:** Multi-variate Cox regression analyses of the 11 of 495 genome instability-related lncRNAs associated with overall survival in NSCLC.

Gene symbol	Coefficient	Hr	HR.95L	HR.95H	*p* value
SCAT1	0.17433805	1.190458	1.063636	1.332402	0.002418
AC002401.4	0.07444835	1.07729	1.025628	1.131554	0.002986
AL079303.1	−0.3116204	0.732259	0.566078	0.947227	0.017654
AL121761.1	−0.0916075	0.912463	0.842924	0.987739	0.023514
TM4SF19-AS1	0.16964836	1.184888	1.011517	1.387974	0.035567
AC027288.1	−0.1709556	0.842859	0.700588	1.014021	0.069932
AC019117.3	−0.0533313	0.948066	0.893767	1.005664	0.076348
AC079949.2	0.09829448	1.103288	0.984168	1.236824	0.091756
AC026369.3	−0.1876336	0.828918	0.660027	1.041026	0.106508
AL355472.2	0.11636521	1.123406	0.961696	1.312308	0.142259
MMP2-AS1	−0.1811897	0.834277	0.654248	1.063845	0.14403

**FIGURE 5:**
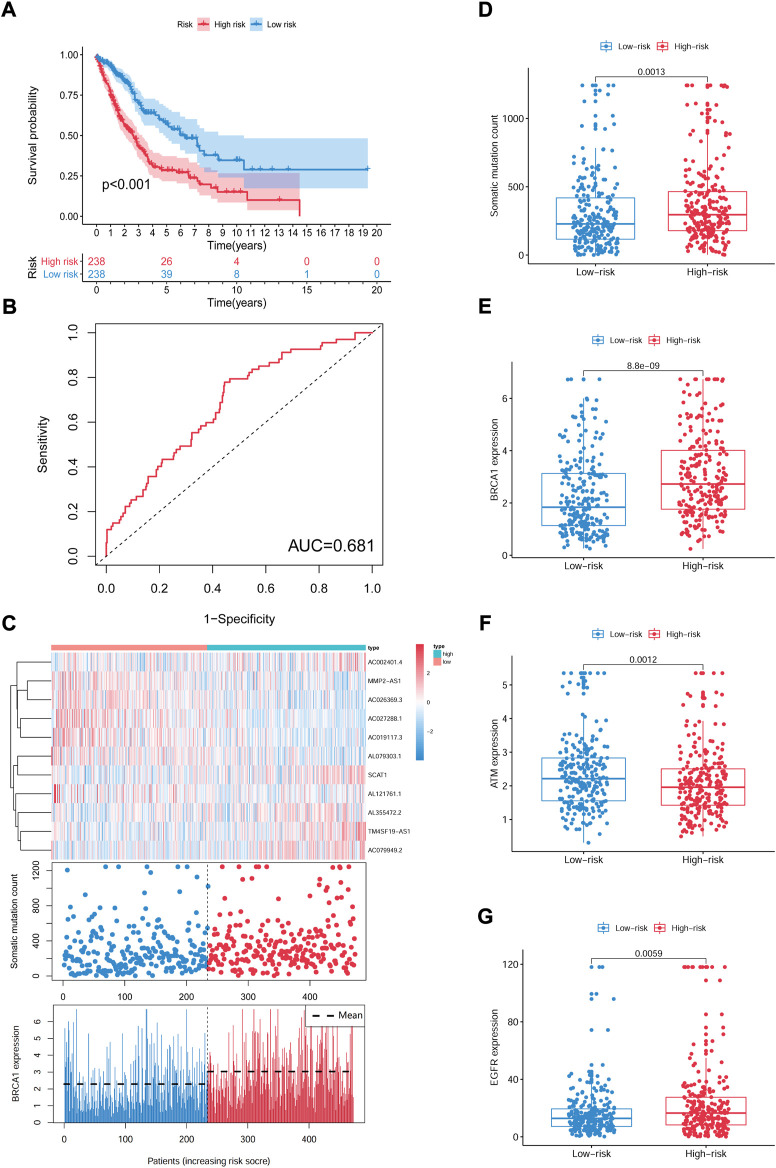
Identification of the GIrLncSig that predicts outcome in the train set. **(A)** Kaplan-Meier estimates of OS for patients predicted by GIrLncSig in the train set. **(B)** ROC curves analysis of GIrLncSig over time. **(C)** LncRNA expression patterns, somatic mutation count and BRCA1 expression in two groups. **(D)** Somatic mutation count in two groups. **(E) (F) (G)** Expression of BRAC1, ATM, and EGFR in two groups. Red stands for the high-risk group and blue for the low-risk group. GIrLncRNAs, genomic instability-related lncRNAs signature; ROC, receiver operating characteristic.

In the equation of GIrLncSig, five lncRNAs (SCAT1AC002401.4TM4SF19-AS1, AC079949.2, AL355472.2) have positive coefficient, implicating that their overexpression correlates with shorter survival, while six lncRNAs (AL079303.1, AL121761.1, AC027288.1, AC019117.3, AC026369.3, MMP2-AS1) have negative coefficient implicating that they are protective factors. Patients with scores above the median GIrLncSig score of 1.065 in the train set were categorized in the high-risk group and vice versa in the low-risk group. Low-risk NSCLC patients survived longer (*p* < 0.001, log-rank test; Figure 5A). The 1-year survival prediction ROC curve for GIrLncSig in the train set had an AUC of 0.681. (Figure 5B). We categorized the patients in the train set by scores and examined the GIrLncSig expression levels, the number of somatic mutations and BRCA1 expression levels in relation to the score (Figure 5C). The expression level of risky lncRNAs SCAT1, AC002401.4, TM4SF19-AS1, AC079949.2, AL355472.2 were upregulated in high-scoring patients, while the protective lncRNAs AL079303.1, AL121761.1, AC027288.1, AC019117.3, AC026369.3, MMP2-AS1 were downregulated. On the contrary, the GIrLncSig in high-scoring patients showed opposite expression patterns. Comparison analysis revealed significant differences between the two groups in somatic mutation counts and BRCA1 expression. Figure 5D shows that a significantly higher somatic mutation count were found in patients in the high-risk group. (*p* = 0.0013, Figure 5D). Furthermore, high-risk patients also had higher BRCA1 and EGFR expression levels (*p* < 0.01, Figures 5E,G). ATM expression levels were lower in high-risk patients (*p* = 0.0012, Figure 5F).

### Independent validation of genomic instability-related long non-coding RNA signature in the non-small cell lung cancer data set

**FIGURE 6:**
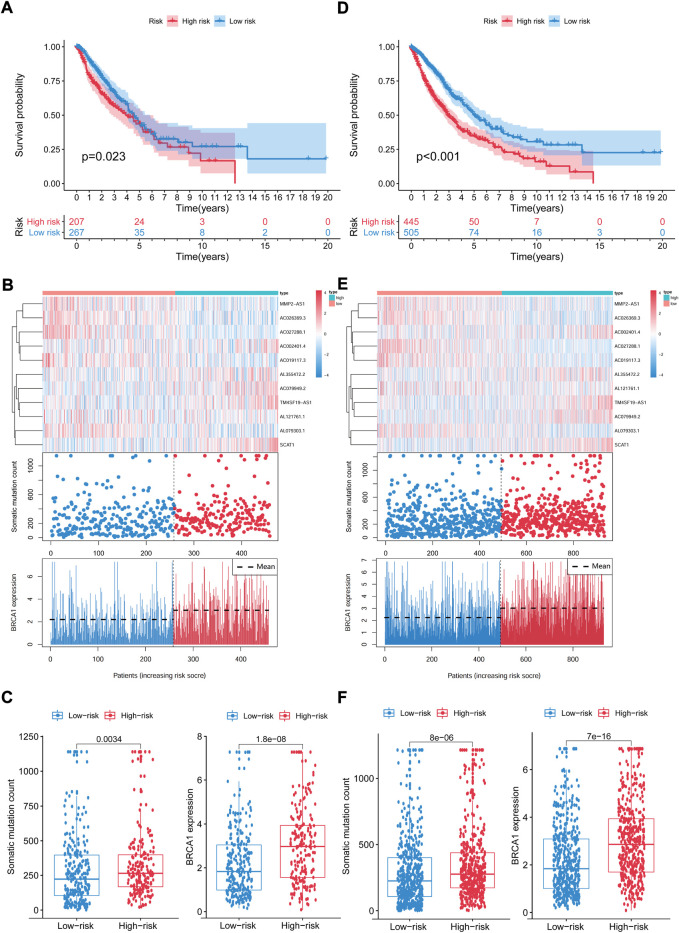
Capability validation of the GIrLncSig in the TCGA set. Kaplan-Meier estimation of overall survival for low- or high-risk patients in the test set **(A)** and TCGA set **(D)** as forecasted by the GIrLncSig. The expression of GIrLncSig, the somatic mutation count and BRCA1 expression among the high- and low-risk groups in the test set **(B)** and TCGA set **(E)**. Number of somatic mutations and BRAC1 expression in high- and low-risk groups in the test set **(C)** and TCGA set **(F)**. Horizontal lines represents median values. The Mann–Whitney U test was used for statistical analysis. GIrLncRNAs, genomic instability-related lncRNAs signature.

We computed the GIrLncSig scores for the test set and the TCGA set, then plotted the ROC curve to validate the survival prediction capability of GIrLncSig. In the test set, the median risk score for GIrLncSig was 1.065. In the test set, patients in the low-risk group had better survival outcomes than those in the high-risk group (*p* = 0.023, Figure 6A). Analogous results were also observed for the entire TCGA set (*p* < 0.001, Figure 6D). Figure 6A showed the expression of GIrLncSig, the somatic mutation count and the expression of BRCA1 in the test set. There were significant differences in somatic mutation patterns between high- and low-risk patients. As shown in Figure 6C, somatic mutation count of patients in the high-risk group is significantly higher compared to that of patients in the low-risk group (*p* = 0.0034, Mann–Whitney U test; Figure 6C). Significantly higher levels of BRCA1 expression were observed in the high-risk group. (*p* < 0.001, Figure 6C).

The prognostic results of the GIrLncSig in the TCGA set were comparable to those described above. Patients in the TCGA set were divided into the high-risk group (*n* = 445) and low-risk group (*n* = 505), where patients in the high-risk group had a shorter median survival than those in the low-risk group (1.5 versus 1.9 years, *p* < 0.001, Figure 6D). The 5-years follow-up survival rate was 11.24% in the high-risk group, which was lower than the 14.65% of the low-risk patients. Figure 6E illustrates the expression pattern of GIrLncSig, the difference in the number of somatic mutations between the two groups and the expression of BRCA1 in the TCGA samples. There was a consequential difference in the number of somatic mutations between the high and low risk groups (*p* < 0.001, Figure 6F). The BRCA1 expression levels in the high-risk group were markedly higher. (*p* < 0.001, Figure 6F).

### Validation of genomic instability-related long non-coding RNA signature as prognostic model

**FIGURE 7:**
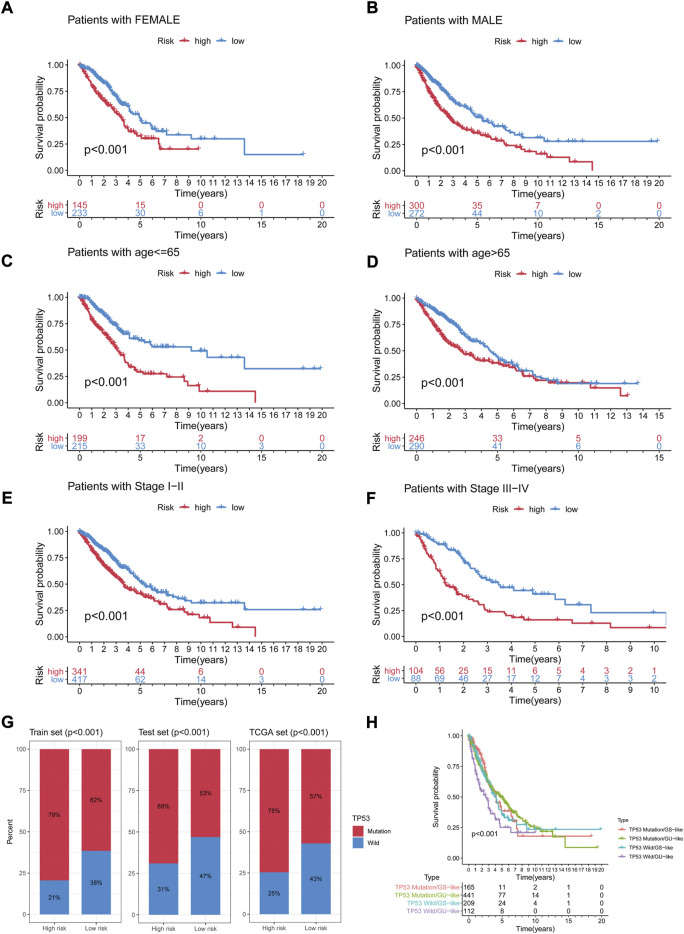
Stratification analyses by age, gender and TNM stage and relationship between the GIrLncSig and TP53. Differences in OS between two groups in female **(A)** samples and male samples **(B)**; in younger samples **(C)** and older samples **(D)**; in early-stage samples **(E)** and late-stage samples **(F)**. **(G)** The percentage of TP53 mutation in the two risk groups in the three sets. **(H)** Kaplan-Meier curve analysis of OS for the four risk groups patients. GIrLncRNAs, genomic instability-related lncRNAs signature; OS, overall survival.

To assess whether the GIrLncSig was an independent clinical variable, multivariate Cox regression analyses were performed on age, gender, TNM stage and risk score. In the multivariate analyses, after adjusting for age, gender and TNM stage, we found that GIrLncSig was remarkably related to overall survival for each set of data. (Table 3). Except for the GIrLncSig, we found age, gender and TNM stage, were also significant. Stratified analysis was used to identify the independence of the prognostic value of GIrLncSig. First, we divided patients in the TCGA set into female (*n* = 378) and male (*n* = 572) groups, then further classified them into high- or low-risk group using the GIrLncSig. In both gender patient groups, overall survival differed significantly between the two groups (*p* < 0.001, Figures 7A,B). We also stratified patients in the TCGA set into a younger patients (*n* = 414) and an older patients group (*n* = 536) according to age≤65 years and >65 years, then allocated them to high- or low-risk group. In the younger patients group and the older patients group, overall survival differed significantly between the high- and low-risk groups (*p* < 0.001, Figures 7C,D). And then, we stratified all patients by pathological stage, with patients with stage I or II combined into the early stage group (*n* = 758) and those with stage III or IV into the late stage group (*n* = 192). As for overall survival, there was a significant difference between the high-risk (*n* = 341) and low-risk (*n* = 417) groups in the early phase group. (*p* < 0.001, Figures 7E). Overall survival also differed significantly in high-risk group (*n* = 104) and low-risk group (*n* = 88) (*p* < 0.001; Figures 7F). These results indicated that the GIrLncSig is an independent predictive sign in NSCLC patients and correlates with overall survival.

**TABLE 3:** Univariate and Multivariate Cox regression analysis of the GIrLncSig and overall survival in different patient sets.

**Variables**	**Univariable model**			**Multivariable model**		
	**Hr**	**95% CI**	***p*** ** Value**	**Hr**	**95% CI**	***p*** ** Value**
Training set (*n* = 476)						
Age	1.007	0.990–1.022	0.406			
Gender	1.211	0.906–1.618	0.195			
Stage	1.364	1.179–1.576	<0.001	0.927	0.658–1.304	0.663
T	1.471	1.239–1.746	<0.001	1.390	1.105–1.747	0.005
M	2.003	1.139–3.520	0.016	2.121	0.864–5.205	0.101
N	1.282	1.071–1.533	0.007	1.187	0.873–1.614	0.274
RiskScore	1.726	1.531–1.946	<0.001	1.682	1.485–1.905	<0.001
Testing set (*n* = 474)						
Age	1.020	1.003–1.036	0.015	1.021	1.005–1.038	0.010
Gender	1.139	0.843–1.538	0.397			
Stage	1.545	1.314–1.815	<0.001	1.302	0.886–1.913	0.178
T	1.357	1.128–1.633	0.001	1.158	0.914–1.465	0.225
M	2.720	1.331–5.555	0.006	1.464	0.542–3.945	0.452
N	1.461	1.210–1.763	<0.001	1.134	0.797–1.612	0.485
RiskScore	1.055	1.009–1.101	0.017	1.058	1.010–1.108	0.017
TCGA set (*n* = 950)						
Age	1.014	1.002–1.024	0.019	1.017	1.005–1.028	0.004
Gender	1.181	0.959–1.454	0.117			
Stage	1.439	1.292–1.602	<0.001	1.177	0.915–1.514	0.203
T	1.410	1.242–1.599	<0.001	1.236	1.053–1.451	0.009
M	2.261	1.453–3.517	<0.001	1.532	0.785–2.988	0.210
N	1.358	1.192–1.545	<0.001	1.155	0.921–1.448	0.212
RiskScore	1.079	1.049–1.108	<0.001	1.082	1.051–1.113	<0.001

As an independent prognostic factor in NSCLC, TP53 is known to maintain genomic stability, whose mutations are associated with worse survival. We further tested whether the predictive performance of GIrLncSig is better than the TP53 mutation status. As shown in Figures 7G, in the three sets, in the high-risk group, TP53 mutations were significantly more common than in the low-risk group. In the train set, a total of 188 patients (79%) in the high-risk group had TP53 mutations, which was significantly higher than 148 patients (62%) in the low-risk group (*p* < 0.001). The number of patients with TP53 mutations detected in the high-risk group was 143 (69%) compared to 142 (53%) in the low-risk group in the test set (*p* < 0.001). In the TCGA set, the number was 334 (75%, high-risk group) and 288 (57%, low-risk group), respectively (*p* < 0.001). Figures 7H manifested the survival curves of the TP53 mutation/GS-like group, TP53 mutation/GU-like group, TP53 wild/GS-like group and TP53 wild/GU-like group (survival rate at 5 years 6.67% versus 17.46% versus 11.48% versus 7.14%, *p* < 0.001). We can deduce that binding to the GIrLncSig may be a better predictor of clinical outcome than TP53 mutation status alone.

### The difference in immune cell infiltration

**FIGURE 8:**
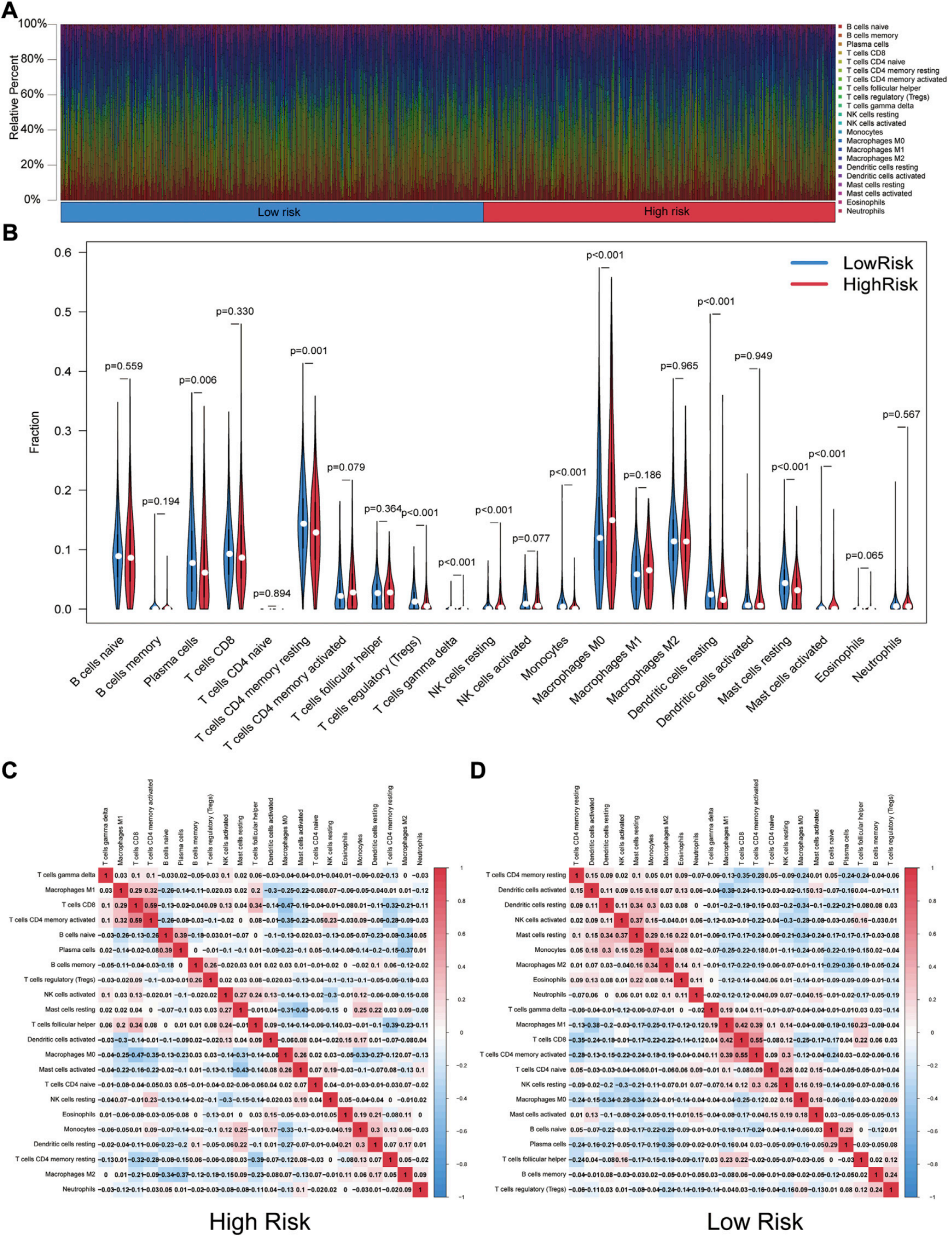
Landscape of tumor-infiltrating immune cells. **(A)** Immune status in the two risk groups. **(B)** Different levels of infiltration of 22 immune cells in the high- and low-risk groups. **(C) (D)** Correlation between immune cells in high- and low-risk groups (red squares represent positively correlated, blue squares represent negatively correlated).

Through the CIBERSORT algorithm, discrepancies in the components of 22 types of tumor-infiltrating immune cells were identified between high- and low-risk patients in NSCLC. Figure 8A summarizes the percentage of immune cells obtained from 950 patients in TCGA. Figure 8B depicts the discrepancies in immune cell infiltration between the two groups. As for correlation between immune cells, for high-risk patients, the proportion of T-cells gamma delta (γδ T-cells), NK cells resting, mast cells activated, and macrophage M0 was significantly higher (Figure 8C). Proportions of plasma cells, T-cells CD4 memory resting, T-cells regulatory (Tregs), dendritic cells resting, monocytes, and mast cells resting were found higher in low-risk patients (Figure 8D).

### Immune checkpoint and chemokines expression in the two risk groups

**FIGURE 9:**
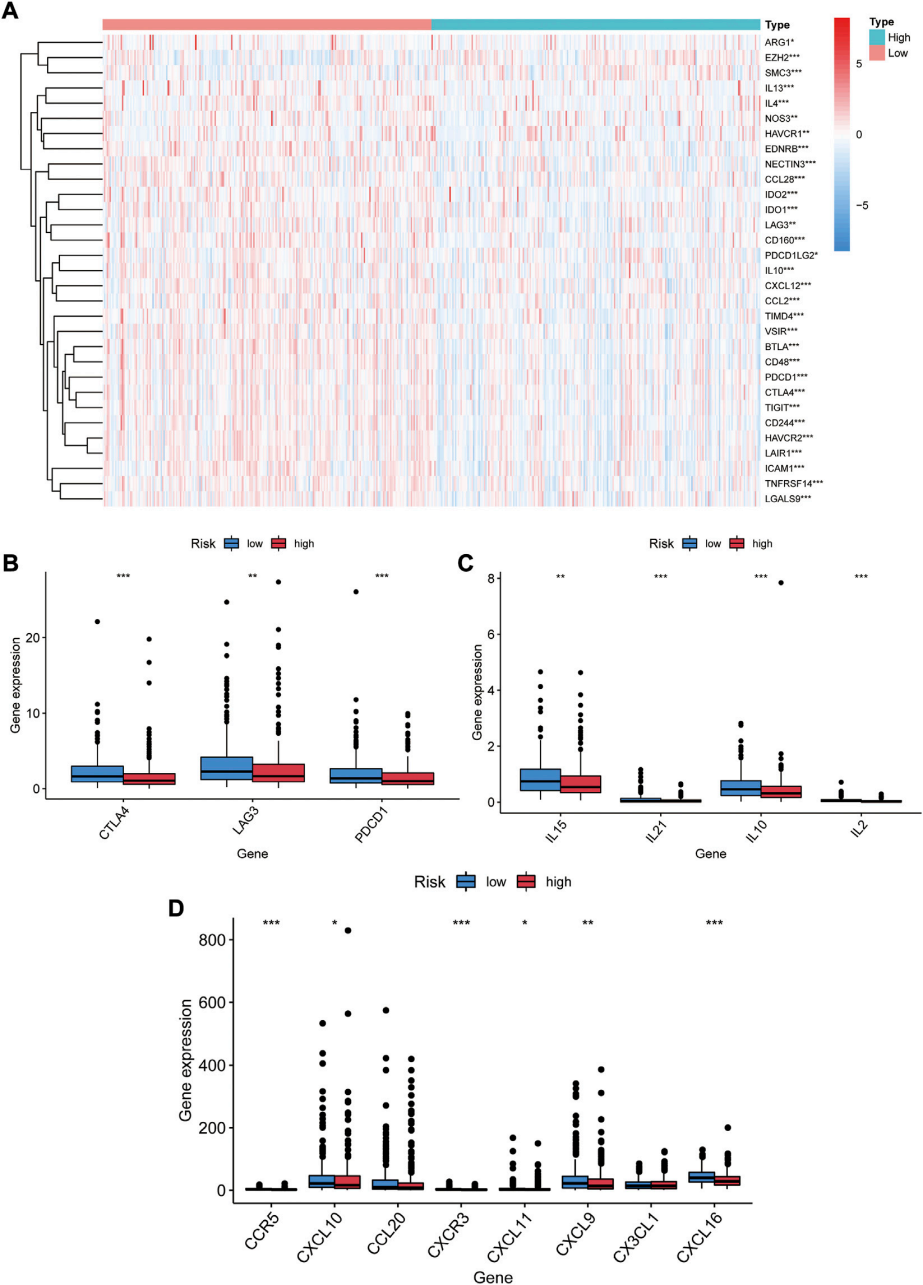
Immune checkpoints and chemokines expression in both risk groups. **(A)** Heat map of gene profiles associated with tumor-promoting effects of both groups in the TCGA database. **(B)** Expression of CTLA-4, LAG3, PDCD1 in two groups. **(C)** Expression of IL15, IL21, IL10, IL2 in two groups. **(D)** Expression of CCR5, CXCL10, CCL20, CXCR3, CXCL11, CXCL9, CX3CL1, and CXCL16 in two groups. *p < 0.05, **p < 0.01, ***p < 0.001.

We examined the exposure of genes associated with tumor-promoting effects in both risk groups. Gene signatures were downloaded from Tracking Tumor Immunophenotype website (http://biocc.hrbmu.edu.cn/TIP/index.jsp). As shown in Figure 9A, genes associated with tumor-promoting effects were mostly downregulated in the high-risk group and upregulated in the low-risk group.

We investigated the expression of immune checkpoints and chemokines in the high- and low-risk groups. Our results showed that PDCD1 were downregulated in the high risk group (*p* < 0.001, Figure 9B). As well, CTLA-4 and LAG-3 expression was markedly lower (*p* < 0.01, Figure 9B). Downregulation of immunosuppressive cytokines (IL15, IL21, IL10, and IL2) was also found in the high-risk group (*p* < 0.01, Figure 9C). Additionally, the expression of immune-activated chemokines (CCR5, CXCL10, CXCR3,CXCL11, CXCL9, and CXCL16) was substantially lessened in the high-risk group than in the low-risk group (*p* < 0.05, Figure 9D). These results suggest that low-risk-score patients tend to develop upregulation of immune checkpoints and chemokines, resulting in an immunosuppressive microenvironment.

### External validation of genomic instability-related long non-coding RNA signature with other long non-coding RNA signatures

**FIGURE 10:**
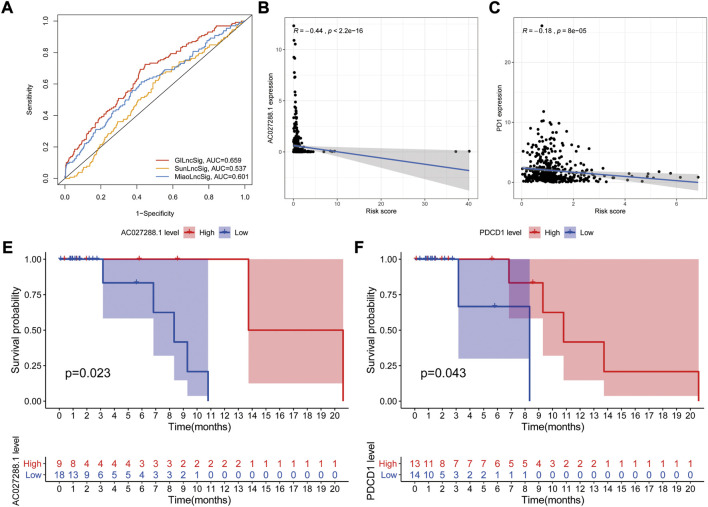
Evaluation of lncRNA signature performance. **(A)** ROC analyses for GIrLncSig, SunGIrLncSig and MiaoGIrLncSig. **(B)** Expression analysis of AC027288.1 in high groups. **(C)** Expression analysis of PD1 in high groups. **(E–F)** Independent validation of the GlrLncRNA. Kaplan-Meier curves showed that OS was worse for patients with low expression levels. GIrLncRNAs, genomic instability-related lncRNAs signature; ROC, receiver operating characteristic; SunGIrLncSig, Sun lncRNA signature; MiaoGIrLncSig, Miao lncRNA signature; OS, overall survival.

A number of lncRNA signatures for predicting prognosis in NSCLC have been published recently. Sun published a lncRNA signature including 7 lncRNAs (Sun et al., 2020a). Miao developed a predictive signature including 8 lncRNAs (Miao et al., 2019). Based on ROC curve analyses, the AUCs of SunGIrLncSig, MiaoGIrLncSig and our GIrLncSig were 0.537, 0.601, and 0.659 (Figure 10A), respectively. The result suggested that our GIrLncSig may performed better than the two published lncRNA signatures in terms of OS prediction.

In order to explore the role of key genes and tumor immune cells played during prognosis of NSCLC, we selected one of GIrLncSigs AC027288.1 and one of immune checkpoints PD1 (PDCD1) for example to performed correlation analysis between gene expression and GIrLncSig risk score in TCGA NSCLC samples. The result shows that the AC027288.1 gene (*R* = −0.44, *p* < 0.001, Figure 10B) and PDCD1 (*R* = −0.18, *p* < 0.001) displayed a negative correlation with risk score, which indicating that AC027288.1 and PDCD1 might both act as protection in the prognosis of NSCLC (Figure 10C).

We further investigated the prognostic value of AC027288.1 from a separate dataset of NSCLC patients treated with anti-PD-1/PD-L1, GSE135222 (*N* = 27) on the GPL16791 Illumina HiSeq 2,500 (Homo sapiens) platform. Patients with high AC027288 expression levels had better OS, suggesting that AC027288.1 may have a protective effect, which was a same result as TCGA dataset. (*p* = 0.023, Figure 10E). This result suggests that patients with high expression levels of AC027288.1 may respond more to PD-1/PD-L1 inhibitors. We also found that patients with high levels of PDCD1 expression lived longer, indicating that PDCD1 may be protective in the prognosis of NSCLC patients treated with anti-PD-1/PD-L1 (*p* = 0.043, Figure 10F).

### Genomic instability-related long non-coding RNA signature was predictive to chemotherapy and molecular targeted therapy response

**FIGURE 11:**
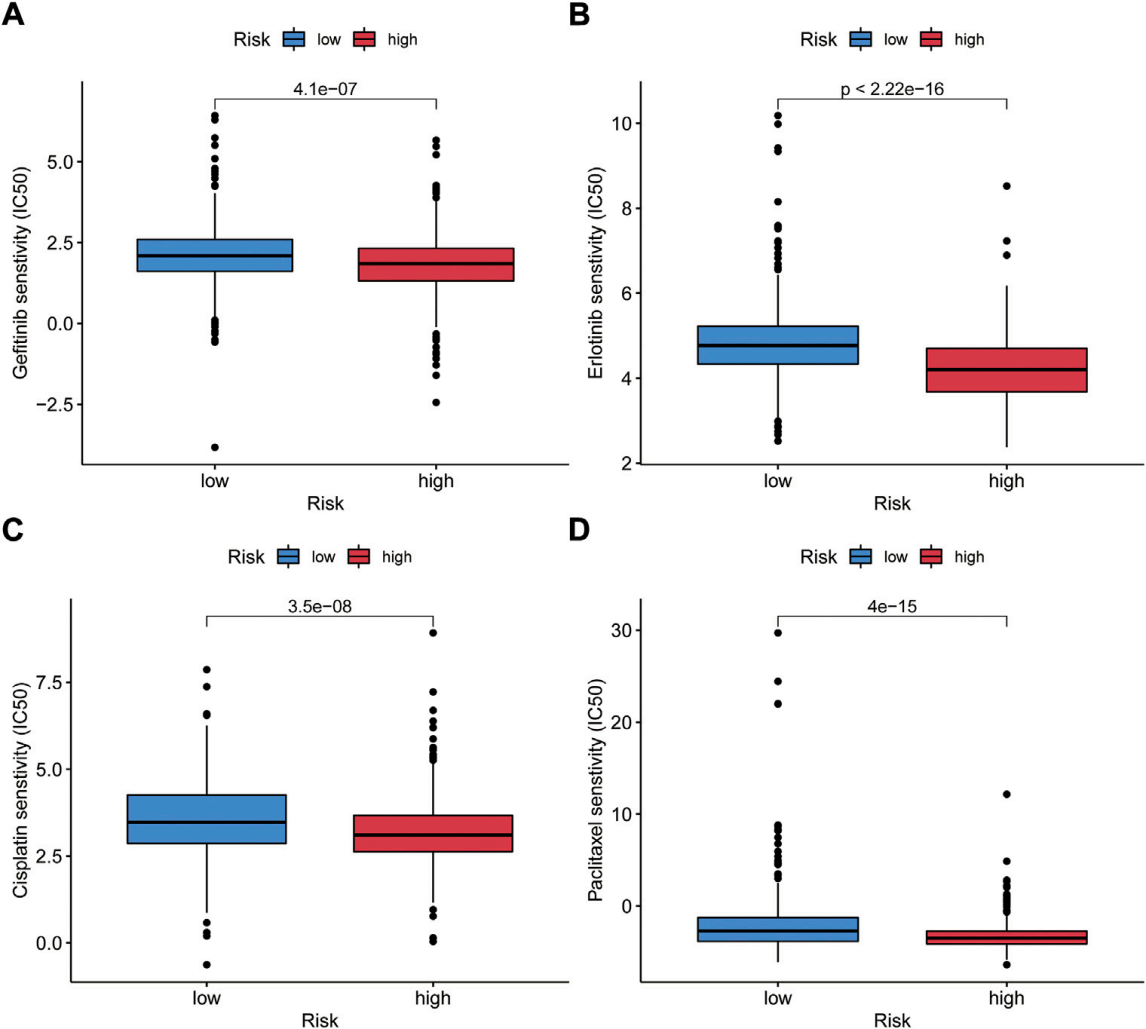
Validation of the relationship between GIrLncSig and the sensitivity of general chemotherapy and targeted therapy. IC50 was calculated for gefitinib **(A)**, erlotinib **(B)**, cisplatin **(C)** and paclitaxel **(D)** in two groups. IC50, the half-maximal inhibitory concentration.

The relationship between risk score and response to chemotherapeutic agents and targeted drugs was investigated. We compared the estimated half-maximal inhibitory concentrations (IC50) of cisplatin and paclitaxel in low- and high-risk patients *via* the pRRophetic algorithm. We have also used this method to study gefitinib and erlotinib, the first-generation targeted drugs used to treat NSCLC. We found that patients in both risk groups had significantly different sensitivities to gefitinib, erlotinib, paclitaxel, and cisplatin (Figures 11A−D). This result suggests that high-risk score associates with increased sensitivity to chemotherapy and molecular targeted therapy. Thus, this prognostic model is effective to forecast the sensitivity of NSCLC patients to these four drugs.

## Discussion

GI is a momentous initiating feature of tumors and promotes tumor progression toward malignancy (Hanahan and Weinberg, 2011). Hereditary cancer susceptibility syndromes, such as Lynch syndrome, are closely related to mutations in DDR genes, and preliminary evidence indicating the significance of GI in tumorigenesis (Li and Martin, 2016). Downregulation or deficiency of the DDR pathway may entail somatic mutations or chromosomal rearrangements, which in turn lead to GI and tumor advancement (Jiang et al., 2021c). DDR genes such as breast cancer susceptibility gene 1/2 (BRCA 1/2), ataxia-telangiectasia mutated (ATM), and BRCA1-associated protein 1 (BAP1) were identified germline or somatic inactivation in 63.5% biliary tract cancer patients (Chae et al., 2019a). TIME is characterized by abnormal blood vessel growth and hypoxia, and its main components include tumor cells and many immune cells such as T lymphocytes, dendritic cells, and tumor-associated fibroblasts (Dai et al., 2017). Tumors can affect the DDR pathway by TIME (Reynolds et al., 1996). Multiple complex interactions between cancer cells and TIME in causing tumor development, and TIME functions in malignant transformation by negatively regulating genome stability by inhibiting the DDR pathway (Yuan et al., 2000; Tlsty and Coussens, 2006). The DNA damage signaling pathway serves an important regulator role for PD-L1 expression upregulation, as DNA damage can increase its expression on the cell surface (Vendetti et al., 2018; Permata et al., 2019). DRR deficiency such as BRCA deletion can significantly upregulate PD-L1 expression (Sato et al., 2017). Studies have demonstrated that in NSCLC and advanced urothelial carcinoma, pathogenic DDR mutations are associated with improved clinical benefit in patients receiving PD-(L) 1 inhibitor therapy (Teo et al., 2018; Ricciuti et al., 2020). Therefore, identifying and characterizing GI and gene mutation status in cancer is of great significance for novel tumor immunotherapy strategies. LncRNAs have received considerable attention for their novel functions in tumor progression, and their aberrant expression may be an essential cause of various diseases in humans, thus highlighting the potential of lncRNAs as diagnostic and prognostic markers (Yang et al., 2019). The human genome contains thousands of lncRNAs, among which DNA damage-activated noncoding RNAs (NORAD, also known as LINC00657) (Elguindy and Mendell, 2021) and GUARDIN (Sun et al., 2020a) are obligate in maintaining genomic stability in human cells. However, the recognition of GI-related lncRNAs for use as diagnostic and prognostic markers is still in its infancy (Bao et al., 2020).

Our study is the first to reveal the lncRNA signatures associating with GI and TIME as prognostic markers in NSCLC patients. Statistically meaningful differences showed in the expression of ATM, BRCA1, PDCD1, and EGFR among the GU-like group and GS-like group. Chromosomal aberrations arising from DNA double-strand breaks (DSBs) are the most pivotal DNA damage in malignant transformation (Zhou and Elledge, 2000). BRCA1 and ATM are key proteins that repair DNA DSBs and control genome integrity (Grabsch et al., 2006). PDCD1 (encoding PD-1 protein) is a key mediator in regulating T-cell activation and tumor antigen priming of TIME (Santarpia et al., 2020), also closely interlinked with TMB (Miao et al., 2020). After treatments with a PD-(L) 1 inhibitor, hyper-progression tends to occur in NSCLC patients with evident tumor cell-intrinsic PD-1 expression. Hence PD-1 has anti-tumor effects in NSCLC (Boussiotis, 2016).

We established the co-expression network of lncRNA-mRNA and performed functional enrichment analysis. PPAR-γ is involved in DDR by promoting ATM signaling (Li et al., 2019). Cytokines regulate the immune function of B lymphocytes, T lymphocytes and natural killer cells by activating intracellular signaling cascades by combining with their specific receptors expressed on the surface of lymphocytes (Hunter and Reiner, 2000). What’s more, Cytokine-cytokine receptor expression on lymphocytes is closely associated with prognosis in relapsed childhood acute lymphoblastic leukemia (Wu et al., 2005). Defects in the DNA mismatch repair pathway lead to a “mutant” cellular phenotype characterized by elevated genomic instability and increased microsatellite instability (MSI), resulting in susceptibility to hereditary nonpolyposis colorectal cancer (Huang and Zhou, 2021).

We further examined predictive power of the 496 GI-related lncRNAs on prognosis of NSCLC patients and constructed the GIrLncSig comprising 11 GI-related lncRNAs (SCAT1, AC002401.4, AL079303.1, AL121761.1, TM4SF19-AS1, AC027288.1, AC019117.3, AC079949.2, AC026369.3, AL355472.2, and MMP2-AS1). We found that 3 lncRNAs have appeared in previous studies among GIrLncSig. In line with the results of other research, SCAT1 was a risk factor and the upregulation of expression was seen to implicate a poor outcome. The downregulation of SCAT1 in A549 cells inhibits the cell proliferation, cell cycle halt at G1 phase, and promoted cellular apoptosis. In addition, an investigation revealed SCAT1 as common independent prognostic biomarkers for LC, the higher expression of SCAT1 in NSCLC correlates with the poor clinical outcomes (Ali et al., 2018). The lncRNA TM4SF19-AS1 is highly consistent with the expression and localization of its host gene TM4SF19, and is considered to be a marker lncRNA of effector T-cells, involved in cell adhesion, regulation of tumor necrosis factor biosynthesis and other related processes of CD8 and CD4 effector T-cells (Luo et al., 2021). MMP2-AS1 is a protective factor and relevant to autophagy-related genes. As a potential treatment target, MMP2-AS1 affects the proliferation, invasion and migration of renal cell carcinoma (RCC) cells by controlling the miR-34c-5p/MMP2 axis to promote the development of RCC. (Fan et al., 2022). Besides, a study constructed and verified a prognostic risk model of targeting autophagy-related gene (ATG) which contains MMP2-AS1 in NSCLC patients and found that this gene was closely involved in immune modulation in TME. (Jiang et al., 2021a). However, after detailed literature search, the biological functions of AC002401.4, AL079303.1, AL121761.1, AC027288.1, AC019117.3, AC079949.2, AC026369.3, and AL355472.2 still had no relevant reports. However, we discovered that the lncRNA AC027288.1 is on chromosome 12q21.2, a locus known to be a carcinogenesis-associated locus in a previous genome-wide association analysis (Sánchez-Tomé et al., 2015). AC002401.4 is positioned on chromosome 17q21.33, which is thought to predict cancer prognosis (Ruiz-Arenas et al., 2019). AL079303.1 is located in the chromosome 14q13.3 region, which was recently described to be associated with lung cancer (Harris et al., 2011). Located on chromosome 12q21.2, AC026369.3 is known to be a susceptibility locus for lung squamous cell carcinoma in previous reports (Shi et al., 2012; Lieberman et al., 2016). LncRNAs, AL121761.1, AC019117.3, AC079949.2, and AL355472.2, however, were not reported previously before this study. Additional investigations are required to ascertain their function in NSCLC.

Seeking to find the association between the GIrLncSig and immune responses, we measured 22 infiltrating immune cell components separately in the low and high-risk groups by the CIBERSORT algorithm. We found obvious different immune infiltrating in two groups classified by the GIrLncSig. It suggested that the proportion of γδ T-cells, resting NK cells, activated mast cells, and M0 macrophage were infiltrated noticeably in high-risk patients. All of them are cells of the innate immune system and interesting mediators in tumor immunotherapy (Marshall and Jawdat, 2004; Vivier et al., 2011; Miyashita et al., 2021; Ricketts et al., 2021). Activated γδ T-cells and quiescent NK cells play anti-tumor roles by inducing the release of cytotoxic molecules and cytokines such as interferon-γ (IFN-γ) and tumor necrosis factor-α (TNF-α) on cancer cells. (Bryceson et al., 2006; Matei et al., 2006; Uchida et al., 2007). Several reports point out that type I IFN signaling and anti-tumor immunity were induced by BRCA1/2 deletion which causing DSB accumulation and elevated levels of GI (Zhao et al., 2019; Reislander et al., 2020; Tarsounas and Sung, 2020). Conversely, six immune cells were apparently in infiltration, namely plasma cells, resting memory T-cells, regulatory T-cells (Tregs), resting dendritic cells, monocytes, and resting mast cells in low-risk patients. Studies have clearly demonstrated that blocking ATM-related DDR can reverse T-cell senescence and suppressive TIME generated by Tregs and tumor cells, thereby enhancing anti-tumor immunity and immunotherapy (Liu et al., 2018).

Immune checkpoints and some chemokines can reflect the response of immunotherapy. ICIs of PD-1 or PDCD1 and cytotoxic T lymphocyte antigen-4 (CTLA-4) have made great achievements in oncology treatment. (Andrews et al., 2017). Lymphocyte activation gene-3 (LAG3; CD223) is a promising target for cancer immunotherapy because it negatively regulates T-cells and binds to PD1 to mediate exhausted state. (Steven et al., 2016). From our research, we noticed that the expression of PDCD1, CTLA-4, LAG-3, immunosuppressive cytokines and chemokines for immune activation were lower in the high-risk group compared with the low-risk group, which was in agreement with the results of several previous works and further demonstrate the predictability of GIrLncSig. In conclusion, GI is implicated in immune infiltration and prognosis of NSCLC patients.

As we know, immunotherapy is an effective and promising therapy in recent years. However, not everyone can get durable responses and benefit from the immunotherapy which may lead to the serious side effects (Kennedy and Salama, 2020). One of the challenges in cancer immunotherapy is developing pre-clinical models that translate to human immunity, the composition of immune cells in TME, tumor antigens and immune cell suppression all make it difficult (Hegde and Chen, 2020). The hyperresponsiveness or unresponsiveness to tumor immunotherapy may be related to the heterogeneity of TIME which is different in different tumor types, patients and tumor stage. Someone suggested the immunotherapeutic response can be better predicted by analyzing and understanding the unique classes and subclasses of TIME and determining the dominant drivers of cancer immunity (Binnewies et al., 2018). And the study of lncRNA is a promising direction. To cite but one example, low lncRNA TCL6 expression may indicate the worse survival rate, while lncRNA TCL6 positively correlated with TILs infiltration and immune checkpoint molecules. (Zhang et al., 2020). Among our study, lncRNA AC027288.1 was one of novel prognostic lncRNAs in GIrLncSig. We discovered the association between AC027288.1 expression and PD-1 expression. Additionally, similar results were also obtained in the external GEO datasets. The outcome implied that GIrLncSig may be as predictive of therapeutic response to ICI therapy as PD-1.

The DDR pathway protects normal cells from some acquired genomic alterations and monitors the presence of exogenous or endogenous DNA damage (Cleary et al., 2020). Many anti-tumor cytotoxic drugs target the DDR signaling pathway for therapeutic effects. The DDR pathway regulates many mechanisms of cancer cell resistance and sensitivity to these cytotoxic drugs (Jiang et al., 2021c). Our findings supported that GIrLncSig were correlation with both resistance to chemotherapeutic and targeted agents, like gefitinib, erlotinib, cisplatin, and paclitaxel, Thereby, drug response to individualized management for NSCLC patients can be predicted.

Although our research supplied a fresh viewpoint on the affinity between GI and TIME and the prognosis of NSCLC, it still existed insufficiencies and required further examination. Firstly, a larger number of independent data sets and experimental verification are necessary to confirm GIrLncSig to make sure its robustness and replicability. Secondly, the mechanism by which GI and tumor immunity interact with each other remains still obscure and needs further elaboration. Besides, there are some new undiscovered lncRNAs in the GI-lncRNA model, and thus prospective studies in the real world will be desired to understand their mechanism in carcinogenesis and progression of NSCLC and verify its clinical application value.

## Conclusion

This study established a risk prognostic signature containing 11 GI-related lncRNAs, and validates the prognostic value from correlation of risk score, immune infiltration and prediction of drug resistance. What’s more, it is the first study to reveal the lncRNA signatures associating with GI and TIME as prognostic marker. One of the discoveries of our study is that the expression of AC027288.1 may be able to reflect the ICI response. Moreover, The DDR pathway is likely to be a potential pathway to influence the OS of NSCLC patients by activating tumor immune recognition and targeting.

## Data Availability

The original contributions presented in the study are included in the article/Supplementary Material, further inquiries can be directed to the corresponding authors.

## Supplementary material

SUPPLEMENTARY FIGURE S1
Forest plot of 34 lncRNAs associated with prognosis in the train set. Red dots represent risky lncRNAs, while blue dots represent protective lncRNAs. The black bar represents the 95% CI of the hazard ratio. CI, confidence interval.
