# Supplementary figures and images for: Comprehensive analysis of somatic mutator-derived and immune infiltrates related lncRNA signatures of genome instability reveals potential prognostic biomarkers involved in non-small cell lung cancer
Source: Front Genet. 2022 Sep 26;13:982030. doi: 10.3389/fgene.2022.982030 (PMC9548567; doi:10.3389/fgene.2022.982030)

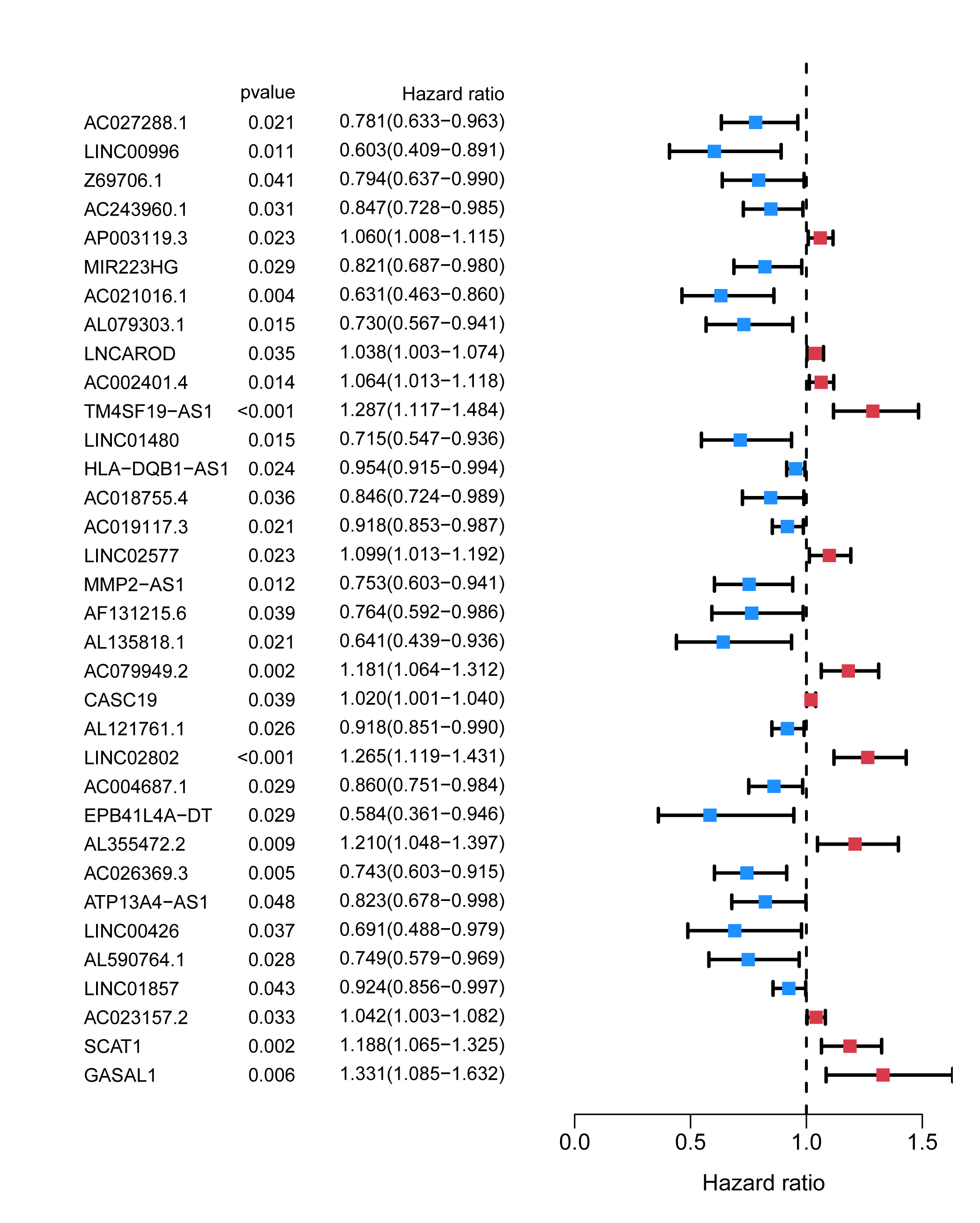

Supplement: Supplementary file 2 [file Image1.TIF]
